# ASPP2 inhibits hepatocellular carcinoma growth by regulating cuproptosis via mitochondrial protein lipoylation

**DOI:** 10.1038/s41419-026-08704-2

**Published:** 2026-04-24

**Authors:** Lu Yang, Xuhui Li, Yutong Jiang, Mingzhu Zhang, Tiantian Wu, Peixian Pi, Zhuozhuo Han, Zhiyu Zhang, Fangyuan Kong, Xiuhong Lu, Hao Yang, Chaohui Zuo, Gang Huang, Jian Zhao, Beibei Liang

**Affiliations:** 1 Shanghai Key Laboratory of Molecular Imaging & Digital and Intelligent Empowerment Biomedical Innovation Center, Pudong Gongli Hospital, Shanghai University of Medicine and Health Sciences, Shanghai, China; 2 Shanghai University of Traditional Chinese Medicine, Shanghai, P.R. China; 3 Research Center of Cancer Prevention and Treatment, Translational Medicine Research Center of Liver Cancer, Hunan Provincial Tumor Hospital, affiliated with the Tumor Hospital of Xiangya Medical School of Central South University, Changsha, China

**Keywords:** Cancer metabolism, Cell death

## Abstract

Hepatocellular carcinoma (HCC) is a metabolically active malignancy. Cuproptosis, a copper-dependent programmed cell death pathway, has been found to be closely associated with tumor progression. Cells with strong oxidative phosphorylation (OXPHOS) capacity exhibit heightened susceptibility to cuproptosis. We demonstrate that the tumor suppressor ASPP2 regulates the key cuproptosis modulator FDX1 by promoting NR2F2 recruitment to the FDX1 promoter in HCC. ASPP2 promotes intracellular copper accumulation through lipoylated protein oligomerization, thereby inducing cuproptosis. Furthermore, ASPP2 facilitates mitochondrial OXPHOS via enhanced tricarboxylic acid (TCA) cycle activity, which sensitizes HCC cells to cuproptosis. In vitro and in vivo experiments confirmed that ASPP2 overexpression suppresses tumor growth and synergizes with copper ionophores. Clinical specimen analysis revealed ASPP2–FDX1 co-expression in 90 HCC cases. A prognostic model incorporating ASPP2 and cuproptosis-related genes demonstrates superior survival prediction in TCGA cohorts. These findings establish the ASPP2–FDX1–NR2F2 axis as a metabolic-cuproptosis link and propose a novel therapeutic strategy for HCC.

## Introduction

HCC is one of the most prevalent and lethal malignancies globally, characterized by a complex pathogenesis involving multifactorial, multistep, and multigenic processes [1, 2]. Despite advancements in diagnostic and therapeutic approaches, the prognosis of HCC remains poor due to its aggressive nature, high metastatic potential, and the emergence of resistance to conventional therapies [3, 4]. Identifying and targeting effective biological pathways are thus pivotal for improving outcomes in HCC patients. Increasing evidence has highlighted the critical role of dysregulated energy metabolism in HCC initiation and progression, presenting a promising therapeutic strategy [5].

Mitochondria play a central role in cellular metabolism, driving key processes such as OXPHOS and the TCA cycle, which are essential for energy production [6]. Recent discoveries have identified a novel form of cell death, cuproptosis, which is copper-dependent and occurs through the accumulation of copper ions in mitochondria [7–9]. The key regulatory protein FDX1 reduces Cu²⁺ to Cu⁺, which directly binds to sulfur-containing mitochondrial proteins, triggering protein aggregation, destabilization of Fe–S cluster proteins, and ultimately leading to proteotoxic stress and cell death [10–12]. This form of cell death provides a unique opportunity to exploit mitochondrial dysfunction in cancer cells, particularly in those exhibiting enhanced OXPHOS activity [13–16].

Emerging research suggests that metabolic plasticity in HCC is further shaped by the dysregulation of tumor suppressor genes. Apoptosis-stimulating protein of p53-2 (ASPP2), a member of the ASPP family, has been identified as a crucial regulator of apoptosis and tumor metabolism [17, 18]. ASPP2 has been shown to inhibit the Warburg effect by suppressing glycolysis and promoting the shift from glycolysis to mitochondrial OXPHOS, thus influencing tumor growth and survival [19]. Notably, recent findings indicate that ASPP2 downregulation in HCC promotes aerobic glycolysis by activating the WNT/β-catenin/HK2 axis, further driving tumor growth and stemness characteristics [9]. Furthermore, ASPP2 is involved in regulating autophagy and energy metabolism, particularly in hepatocellular carcinoma [20]. Downregulation of ASPP2 in HCC has been associated with increased glycolytic activity, reduced mitochondrial OXPHOS, and enhanced tumor survival under stress [21]. This dual role in regulating both energy metabolism and cell death positions ASPP2 as a potential therapeutic target for HCC.

This study aims to investigate the relationship between ASPP2, mitochondrial metabolism, and cuproptosis in the context of HCC. By elucidating how ASPP2 modulates mitochondrial OXPHOS and promotes Cuproptosis, we explore the potential of targeting ASPP2 to sensitize HCC cells to copper-based therapies. Furthermore, we assess the impact of ASPP2 expression on tumor growth and metabolic reprogramming using in vitro and in vivo models. Our findings provide new insights into the role of ASPP2 in regulating mitochondrial function in hepatocellular carcinoma.

## Materials and methods

### Cell culture

High-metastatic human hepatocellular carcinoma cells (HCC-LM3) purchased from Fenghui Biotechnology Co., Ltd. and human hepatocellular carcinoma cells (Huh7) provided by the Eastern Hepatobiliary Surgery Hospital were used. Cells were maintained in DMEM high-glucose medium (Hyclone), supplemented with 10% fetal bovine serum (BI) and 1% penicillin–streptomycin (Absin). The cells were maintained at 37 °C in a humidified atmosphere with 5% CO_2_.

### Quantitative real-time PCR

Total RNA was isolated using the BIOMIGA RNA extraction kit. Reverse transcription was performed using HiScript qRT SuperMix (Vazyme).qPCR was conducted using ChamQ SYBR Color qPCR Master Mix (Vazyme). The primer sequences are in the supplementary materials (Table S1).

### Western blotting

Total protein was extracted using RIPA lysis buffer containing 1% phenylmethylsulfonyl fluoride (Beyotime). Protein concentrations were determined using the Bicinchoninic Acid kit (Yeasen). The protein samples were separated by SDS–PAGE and transferred onto PVDF membranes. Membranes were incubated overnight at 4 °C with primary antibodies and subsequently probed with secondary antibodies at room temperature for 1–2 h. Detection was performed using SuperSignal™ West Pico PLUS chemiluminescent substrate, and images were captured using a BioRad ChemiDocTM.anti-ASPP2 body (SIGMA, A4480), anti-FDX1 body (abcam, ab108257), anti-LIAS body (proteintech, 11577-1-AP), anti-DLAT body (Cell signaling, 12362S), anti-DLST body (abcam, ab177934), anti-PDHA1 body (abcam, ab168379), and anti-PDHB body (abcam, ab155996).

### Plasmid construction and lentiviral transfection

Short hairpin RNAs targeting ASPP2 (LV-shASPP2; 5′-GCTGAGGGAGAAAGAGAAGAA-3′) and a non-targeting control (LV-shNon; U6-shRNA-cmv-tp53bp2) were obtained from Hanbio (Shanghai, China). HCC-LM3 cells were infected with concentrated lentivirus at a multiplicity of infection (MOI) of 20 in the presence of 8 μg/mL polybrene (Sigma). After 24 h, the viral medium was replaced with fresh complete medium. Stable cell lines were selected with puromycin, and knockdown efficiency was confirmed by qRT-PCR and Western blotting.

For ASPP2 overexpression, the full-length human ASPP2 cDNA was cloned into the pcDNA3.1(+) vector (designated pcASPP2, kindly provided by Dr. Xin Lu, Ludwig Institute for Cancer Research, Oxford, UK). Huh7 cells were transfected with pcASPP2 using Lipofectamine™ 3000 (Invitrogen, USA) following the manufacturer’s instructions. Cells transfected with the empty pcDNA3.1(+) vector served as controls.

For FDX1 manipulation, overexpression and knockdown were achieved using pcDNA3.1 vectors, pcFDX1 and siRNA duplexes (GenePharma, China). The siRNA-targeting sequence for FDX1 was 5′-CCUGUCACCUCAUCUUUGATT-3′. Transfections were performed using Lipofectamine 3000 (ShareBio, China) in Opti-MEM (Gibco, USA), and stable transfectants were selected with 4 μg/mL puromycin for 72 h.

### Chromatin immunoprecipitation (ChIP)

HCC-LM3 cells were crosslinked with 1% formaldehyde for 15–30 min at room temperature and quenched with 125 mM glycine. Cells were lysed in SDS buffer with protease inhibitors, and chromatin was sheared to 200–1000 bp fragments using a Bioruptor (Diagenode). For immunoprecipitation, 10 μg of chromatin was incubated with 2–5 μg anti-ASPP2 antibody (Abcam, USA) or control IgG (Beyotime, China) overnight at 4 °C, followed by Protein A/G magnetic beads for 4 h. After washing, complexes were eluted and reverse-crosslinked at 65 °C, then treated with RNase A and proteinase K. DNA was purified using a PCR purification kit (Beyotime, China) and analyzed by qPCR with specific primers (Table S2). Enrichment was calculated relative to the input or IgG control.

### Dual luciferase reporter assay

HCC-LM3 cells were infected with lentiviral shNon or shAspp2 and expanded. Cells were seeded in 48-well plates (3 × 10⁴ cells/well) and co-transfected with firefly luciferase reporter constructs and Renilla luciferase (TK) plasmid as an internal control. After 48 h, cells were lysed, and luminescence was measured using a dual-luciferase assay system. Firefly luciferase activity was normalized and statistical analysis was performed using a two-tailed Student’s *t*-test.

### Immunofluorescence (IF) assay

Cells were rinsed three times with PBS and fixed in methanol for 15 min. Following fixation, they were permeabilized with 0.5% Triton X-100 for 10 min, then washed again with PBS. Non-specific binding was blocked by incubating the cells in 5% BSA at 4 °C overnight. After additional PBS washes, cells were incubated with primary antibodies. Fluorescent images were captured using a Zeiss LSM880 confocal microscope (Carl Zeiss, Germany).

### Patient samples

A tissue microarray was constructed by Shanghai Zhuohao Biotechnology Co., Ltd (catalog numbers LVC160001/LVC160003). The company was responsible for obtaining written informed consent from all participants prior to sample collection. A total of 90 hepatocellular carcinoma (HCC) tumor specimens were included, all obtained from patients who underwent curative hepatic resection between 2008 and 2015. Relevant clinicopathological characteristics of the patients are summarized in Table 1.

**Table 1:** The associations of ASPP2 and FDX1 expression with clinicopathologic characteristics in 90 patients with HCC.

	Whole study (*n* = 90)			ASPP2 negative group (*n* = 58)			ASPP2 positive group (*n* = 32)		
	FDX1 expression		*P*	FDX1 expression		*P*	FDX1 expression		*P*
	Negative (*n* = 46)	Positive (*n* = 44)		Negative (*n* = 36)	Positive (*n* = 22)		Negative (*n* = 10)	Positive (*n* = 22)	
Sex			0.689			1.000			1.000
male	41	38		32	19		9	19	
Female	5	6		4	3		1	3	
Age (years)			0.686			0.777			1.000
<50	26	23		21	12		5	11	
≥50	20	21		15	10		5	11	
HBsAg			0.324			0.357			1.000
Negative	5	8		2	3		3	5	
Positive	41	36		34	19		7	17	
AFP (ng/mL)			0.202			0.536			0.960
≤400	21	26		15	11		6	15	
>400	25	18		21	11		4	7	
Tumor volume (cm^3^)			0.411			0.48			0.308
≤5	18	21		13	10		5	11	
>5	28	23		23	12		5	11	
AJCC Stage			0.018			0.301			0.047
Stage Ⅰ–Ⅱ	11	21		10	9		1	12	
Stage Ⅲ–Ⅳ	35	23		26	13		9	10	
Recurrence time (months)			0.042			0.088			0.018
≤6	24	10		18	6		6	4	
>6	22	24		18	6		4	18	

*P* values are two-tailed and based on the Pearson *χ*^2^ test.

### Cellular glycolysis stress and oxygen consumption assay

Extracellular acidification rates (ECAR) and oxygen consumption rates (OCR) were measured in real time using the Seahorse XF24 Flux Analyzer, following the manufacturer’s instructions. HCC-LM3, Huh7 cells, including ASPP2 knockdown and control cells, were seeded in XF24 plates and allowed to adhere overnight. Sequential injections of oligomycin (1 μM), FCCP (1 μM), and rotenone/antimycin (1 μM) were used to measure OCR. ECAR was determined following the addition of glucose (10 mM), oligomycin (1 μM), and 2-deoxyglucose (80 mM). Data were normalized to cell counts and expressed as pmoles/min for OCR and mpH/min for ECAR.

### Intracellular reactive oxygen species (ROS) production assay

Intracellular ROS levels were measured using the DCFH-DA fluorescent probe according to the manufacturer’s instructions (Beyotime). Cells were incubated with 10 μM DCFH-DA for 20 min at 37 °C, washed three times with PBS, and analyzed by flow cytometry.

### Mitochondrial fractionation, extraction and identification

Mitochondrial and cytosolic fractions were prepared using a mitochondrial isolation kit (Thermo Scientific). Briefly, cells were resuspended in ice-cold mitochondrial extraction buffer containing 1 mM PMSF and homogenized using a glass homogenizer. The homogenate was centrifuged at 600×*g* for 10 min at 4 °C to remove nuclei. The supernatant was further centrifuged at 11,000×*g* for 10 min to collect mitochondrial pellets. The purity of the isolated mitochondria was assessed by Western blotting using specific mitochondrial and cytoplasmic marker proteins. The structural integrity of the mitochondria was further examined by transmission electron microscopy (TEM).

### Cell viability assay

Cell viability was assessed using the CCK-8 assay. Cells were seeded in 96-well plates (1 × 10⁴ cells/well) and treated with Elesclomol (100 nM), tetrathiomolybdate (20 μM), Z-VAD-FMK (30 μM), Ferrostatin-1 (10 μM), Necrostatin-1 (20 μM), or Chloroquine (5 μM) for 48 h. Absorbance was measured at 450 nm using a microplate reader.

### Enzyme activities and copper assay

SDH Activity Assay Kit (Solarbio), PDH Activity Assay Kit (Solarbio), α-KGDH Activity Assay Kit (Solarbio), Citrate synthase assay kit (Nanjing Jiancheng Bioengineering Institute), Micro Mitochondrial complex Ⅰ Activity Assay Kit (Abbkine), Micro Mitochondrial complex Ⅰ Activity Assay Kit (Abbkine) were determined using commercial kits. Intracellular copper levels were determined using a copper colorimetric assay kit (Elabscience, China). Optical density at 580 nm was used to calculate copper concentrations.

### EdU cell proliferation assay

Cell proliferation was evaluated using a 5-ethynyl-2′-deoxyuridine (EdU) incorporation assay (Beyotime, Shanghai, China). Briefly, treated cells were seeded into 96-well plates at a density of 5 × 10³ cells/well and incubated with 50 μM EdU for 2 h at 37 °C. After fixation with 4% paraformaldehyde and permeabilization, nuclei were counterstained with DAPI for 20 min. Images were captured using a fluorescence microscope (Olympus, Tokyo, Japan).

### Immunohistochemistry assay

Following fixation in 10% formalin, tumor tissues were embedded in paraffin blocks. Serial sections (4 μm thickness) were cut and subjected to deparaffinization in xylene, followed by dehydration through a graded ethanol series (100–70%) and rehydration in deionized water. Antigen retrieval was performed by immersing sections in boiling citrate buffer (pH 6.0) for 5 min. Endogenous peroxidase activity was blocked by incubating sections in 3% hydrogen peroxide solution for 25 min at room temperature in the dark. Sections were then developed using a diaminobenzidine (DAB) chromogenic substrate and imaged with a digital microscopy system.

### Tumor xenograft assay

Male BALB/c nude mice, aged 4 weeks, were purchased from the Shanghai Experimental Animal Center of the Chinese Academy of Sciences (Shanghai, China). All animal procedures were approved by the Medical Ethics Committee of Shanghai University of Medicine & Health Sciences(approval number: 2018-GZR-18-310110196803058627). Mice were randomly assigned to four groups (*n* = 5 per group) using a blinded selection process. HCC-LM3 cells (1 × 10⁷), infected with either shNon-Luc or shAspp2-Luc lentivirus, were subcutaneously injected into the flank of nude mice. Tumor volumes were measured every 3 days, and when tumors reached ~100 mm³ in size, mice received tail vein injections of either ES–Cu (5 mg/kg) or PBS (vehicle control) every 48 h for a total duration of 3 weeks. After the treatment ended, all mice were euthanized, and tumor tissues were harvested for immunohistochemical analyses.

### RNA-seq, mitochondrial proteomics, and ATAC-seq

HCC-LM3 cells stably expressing control shNon and shAspp2 were used for transcriptomic, proteomic, and chromatin accessibility analyses. For RNA sequencing, total RNA was extracted using TRIzol® reagent following the manufacturer’s protocol. For ATAC-seq, ~5 × 10⁴ cells per sample were collected and lysed in buffer containing 0.1% NP-40, 0.1% Tween-20, and 0.01% digitonin to isolate nuclei, which were subsequently subjected to transposase tagmentation and library preparation.

### Bioinformatics analysis

Transcriptomic and clinical data of LIHC were obtained from The Cancer Genome Atlas (TCGA) (https://portal.gdc.cancer.gov) and the International Cancer Genome Consortium (ICGC) (https://icgc.org/). All statistical analyses were performed using R software (version 4.4.2). Survival differences were assessed using Kaplan–Meier curves and compared by the log-rank test. The time-dependent receiver operating characteristic (ROC) analysis was applied to evaluate the predictive performance of the models. To identify survival-associated genes, univariate Cox proportional hazards regression was first conducted, followed by least absolute shrinkage and selection operator (LASSO) regression to prevent overfitting and refine variable selection. Subsequently, a multivariate Cox regression model was constructed to establish a prognostic gene signature.

For the single-cell analysis of cuproptosis-related correlations, single-cell RNA sequencing (scRNA-seq) data of hepatocellular carcinoma were retrieved from a publicly available dataset (https://omic.tech/scrna-hcc) [22]. Hepatocytes were categorized into three phenotypic subsets—non-malignant hepatocytes, pro-tumorigenic hepatocytes, and pro-metastatic hepatocytes—according to transcriptional signatures and cell annotation criteria described in the original study. Pairwise gene–gene correlations between ASPP2 and cuproptosis-related genes were calculated within each phenotypic subset using Spearman’s rank correlation. The resulting correlation coefficients (*ρ* values) were visualized as heatmaps using the “corrplot” package in R, with hierarchical clustering applied to uncover global expression patterns. Non-significant correlations (adjusted *p* > 0.05) were omitted from visualization to enhance interpretability.

### Statistical analysis

Data are presented as mean ± SD. Differences between groups were analyzed by Student’s *t*-test or ANOVA using GraphPad Prism 8.0 and SPSS 27.0. Survival curves were compared by the log-rank test. *P*-value < 0.05 was considered statistically significant.

## Results

### ASPP2 enhances mitochondrial metabolism and tricarboxylic acid cycle function

The expression levels of ASPP2 vary among different HCC cell lines [23]. To functionally manipulate ASPP2 levels, we selected Huh7 cells—characterized by relatively low endogenous ASPP2 expression—for plasmid-based overexpression, and HCC-LM3 cells—exhibiting high basal ASPP2 levels—for lentiviral knockdown. Quantitative PCR and western blot analyses confirmed the efficiency of these manipulations (Fig. S1A and B). In Huh7 cells, ASPP2 mRNA levels increased ~1.7-fold and protein levels 1.4-fold upon pcASPP2 transfection compared with control (pcDNA). Conversely, in HCC-LM3 cells, shASPP2 reduced ASPP2 mRNA to ~20% of control levels and protein expression to ~15% of baseline (*P* < 0.001; Fig. S1B). These results verified the successful establishment of ASPP2 gain- and loss-of-function models for subsequent assays.

Mitochondrial stress, which is reflected by the OCR, serves as an indicator of respiratory function. Impaired mitochondria reduce OCR, prompting a metabolic shift to aerobic glycolysis, which increases extracellular acidification. Thus, ECAR reflects glycolytic activity [24, 25].

**Fig. 1:**
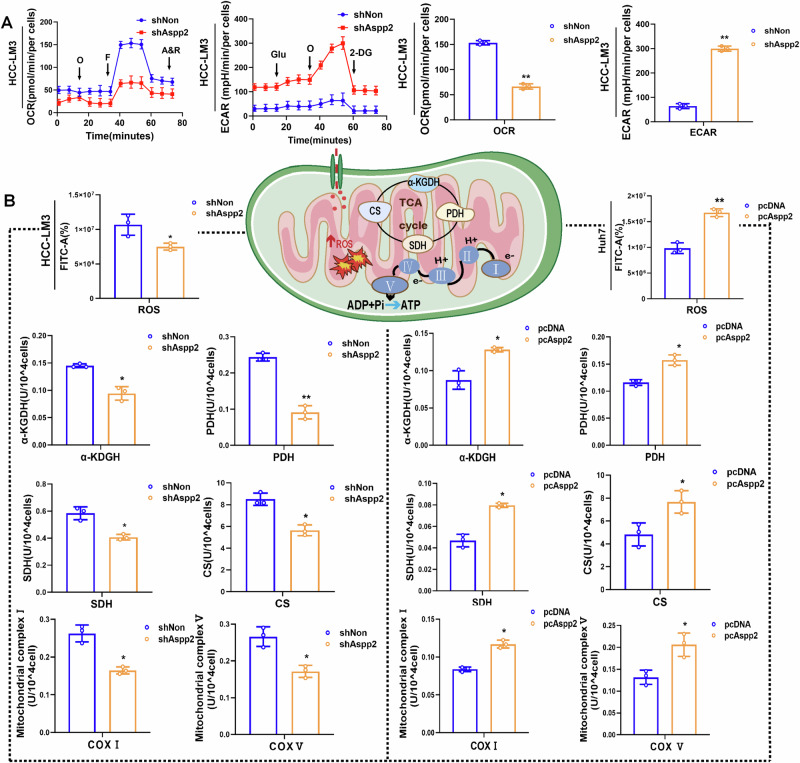
ASPP2 enhances mitochondrial metabolism and tricarboxylic acid cycle function. **A** Measurement of OCR and ECAR in HCC cells with altered ASPP2 expression. **B** Assessment of mitochondrial ROS levels in HCC-LM3 and Huh7 cells with ASPP2 overexpression or knockdown. Enzymatic activity assays of TCA cycle components, including PDH, CS, SDH, and α-KGDH, in HCC-LM3 and Huh7 cells. Measurement of mitochondrial complex I (NADH–CoQ reductase) activity and complex V (ATP synthase) activity in ASPP2-overexpressing and ASPP2-knockdown HCC cells.Data are expressed as mean ± SD (*n* = 3); *p < 0.05, **p < 0.01, ***p < 0.001.

To investigate the role of ASPP2 in mitochondrial metabolism, ECAR and OCR were measured in control and ASPP2-knockdown HCC-LM3 cells using a Seahorse XF24 analyzer after 24 h of culture under standard conditions. Compared to the control group, ASPP2 knockdown significantly increased ECAR while reducing OCR (*P* < 0.01) (Fig. 1A), suggesting that ASPP2 inhibits glycolysis while promoting mitochondrial oxidative phosphorylation.

ROS, generated as byproducts of oxidative phosphorylation, reflect mitochondrial function. Flow cytometry analysis using a ROS detection kit demonstrated that ASPP2 overexpression significantly elevated ROS levels in Huh7 cells (*P* < 0.01), whereas ASPP2 knockdown reduced ROS levels in HCC-LM3 cells (*P* < 0.05) (Fig. 1B), indicating that ASPP2 enhances mitochondrial ROS production.

To further delineate the role of ASPP2 in mitochondrial metabolism, key TCA cycle enzymes were assessed [26]. Pyruvate dehydrogenase (PDH), a critical enzyme facilitating pyruvate entry into the mitochondria, exhibited increased activity in ASPP2-overexpressing Huh7 cells (*P* < 0.05), whereas ASPP2 knockdown reduced PDH activity in HCC-LM3 cells (*P* < 0.01) (Fig. 1B). Similarly, citrate synthase (CS), succinate dehydrogenase (SDH), and α-ketoglutarate dehydrogenase (α-KGDH) activities were significantly elevated following ASPP2 overexpression but decreased upon ASPP2 knockdown (*P* < 0.05) (Fig. 1B), suggesting that ASPP2 enhances TCA cycle activity.

Mitochondrial respiratory chain complexes were also examined to assess oxidative phosphorylation efficiency [27]. Complex I (NADH-CoQ reductase) activity was significantly increased in ASPP2-overexpressing Huh7 cells (*P* < 0.05), whereas ASPP2 knockdown reduced its activity in HCC-LM3 cells (*P* < 0.05) (Fig. 1B), indicating that ASPP2 facilitates mitochondrial electron transport. Likewise, Complex V (ATP synthase) activity followed the same pattern (*P* < 0.05) (Fig. 1B), reinforcing the role of ASPP2 in promoting mitochondrial ATP production.

Collectively, these findings suggest that ASPP2 enhances mitochondrial oxidative phosphorylation while suppressing aerobic glycolysis in hepatocellular carcinoma cells by regulating key metabolic enzymes and electron transport chain components.

### ASPP2 promotes the occurrence of cuproptosis

**Fig. 2:**
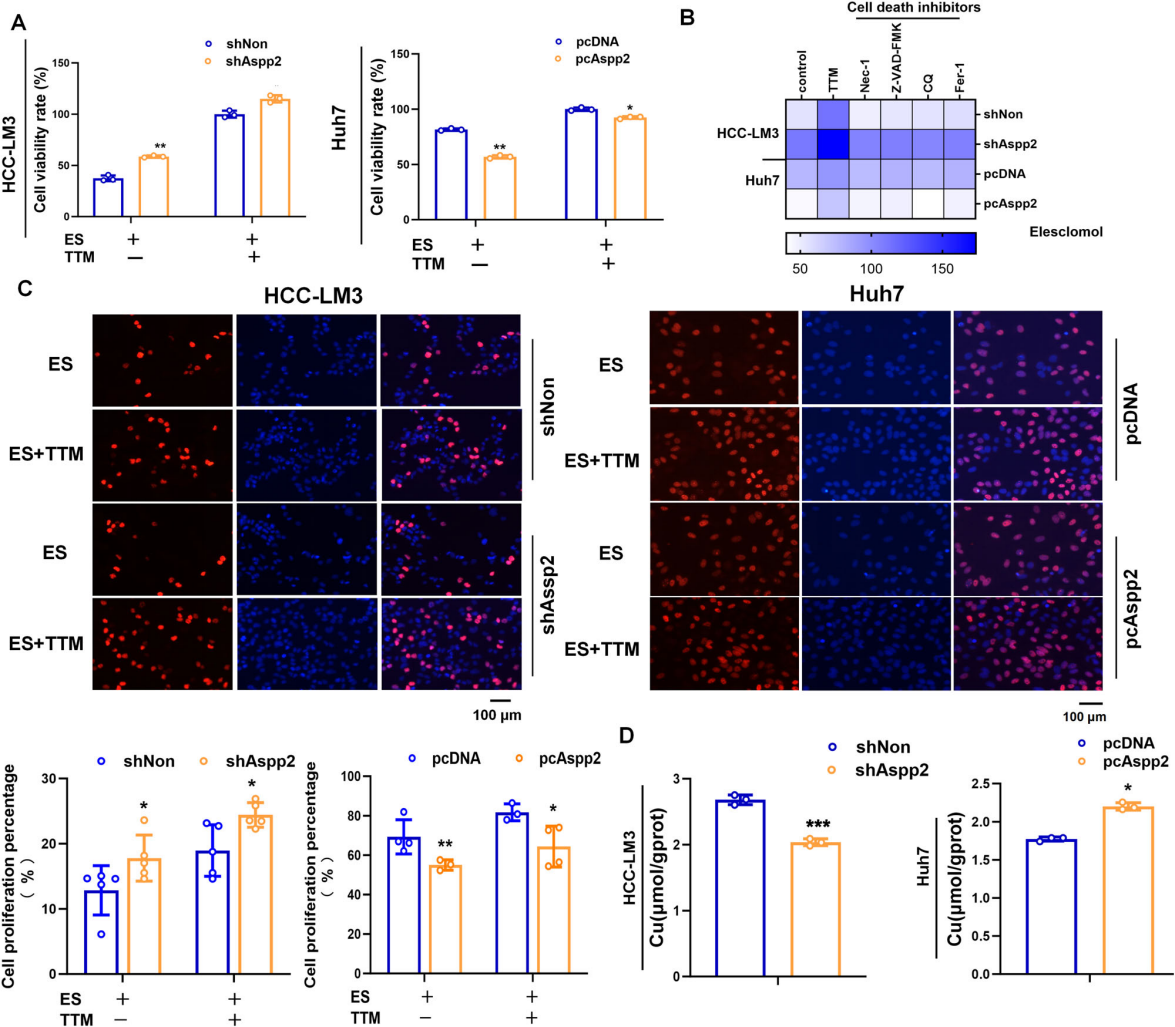
ASPP2 promotes the occurrence of cuproptosis. **A** Cell viability assay assessing the effects of ES treatment with or without the copper chelator TTM in ASPP2-overexpressing Huh7 cells and ASPP2-knockdown HCC-LM3 cells. **B** Heatmap of cell death inhibitor screening (Nec-1, CQ, Fer-1, and Z-VAD-FMK) showing that ES-induced cytotoxicity was unaffected by necroptosis, autophagy, ferroptosis, or apoptosis inhibitors but was rescued by TTM. **C** EdU proliferation assay was performed to detect the proliferation status of ASPP2-overexpressing Huh7 cells and ASPP2-knockdown HCC-LM3 cells. The cells were subjected to the following treatments: ES treatment alone and co-treatment with ES and TTM. Scale bar: 100 μm. **D** Quantification of intracellular copper levels in Huh7 and HCC-LM3 cells after ES treatment. Data are expressed as mean ± SD (*n* = 3); *p < 0.05, **p < 0.01, ***p < 0.001.

Elesclomol (ES) is a copper ionophore that binds extracellular Cu²⁺, transports it into cells, and induces cuproptosis. Tetrathiomolybdate (TTM) is a copper ion chelator that can bind copper ions, reducing intracellular copper levels and inhibiting Cuproptosis [28, 29]. In this experiment, TTM was used as a negative control. The results showed that overexpression of ASPP2 in Huh7 cells, when treated with ES, significantly reduced cell viability compared to the control group, whereas the addition of TTM attenuated this effect (*P < 0.05). In HCC-LM3 cells with lentiviral knockdown of ASPP2, the cell viability after ES treatment was higher than that in the shNon group, but the addition of TTM significantly increased cell viability in both groups (*P < 0.05), especially in the shAspp2 group (Fig. 2A).

To further confirm that ASPP2 promotes Cuproptosis in hepatocellular carcinoma cells rather than other types of cell death, the experiment was conducted with Huh7 and HCC-LM3 cells expressing different levels of ASPP2. After ES treatment, the cells were treated with inhibitors of necroptosis (Necrostatin-1, Nec-1), autophagy (Chloroquine, CQ), ferroptosis (Ferrostatin-1, Fer-1), and apoptosis (Z-VAD-FMK), and cell activity was assessed. The results showed that none of the inhibitors rescued Cuproptosis. However, the addition of the copper chelator TTM significantly increased cell viability (Fig. 2B).

Similarly, EdU proliferation assays demonstrated distinct responses to ES treatment based on ASPP2 expression levels. Huh7 cells overexpressing ASPP2 exhibited significantly lower EdU positivity rates compared to controls, indicating reduced proliferative capacity and enhanced sensitivity to ES-induced cytotoxicity. Conversely, HCC-LM3 cells with lentiviral-mediated ASPP2 knockdown showed attenuated responses to ES, with higher EdU positivity rates. Strikingly, this effect was reversed upon co-treatment with the copper chelator TTM, which restored proliferation rates to near-baseline levels (Fig. 2C). These observations strongly suggest that ASPP2 potentiates ES-mediated growth inhibition through a copper-dependent mechanism, further supporting cuproptosis as the primary mode of cell death.

Measurements of intracellular copper levels corroborated these findings. The copper content in exogenous cells was measured in Huh7 cells overexpressing ASPP2 (pcAspp2, pcDNA) and HCC-LM3 cells with lentiviral knockdown of ASPP2 (shAspp2, shNon) after ES treatment. The results indicated that overexpression of ASPP2 may enhance intracellular copper accumulation, whereas ASPP2 knockdown potentially promotes copper efflux or inhibits copper transport, reducing intracellular copper ion levels (Fig. 2D).

### Mitochondrial proteomics revealed the regulatory role of ASPP2 on cuproptosis-related proteins in hepatocellular carcinoma cells

**Fig. 3:**
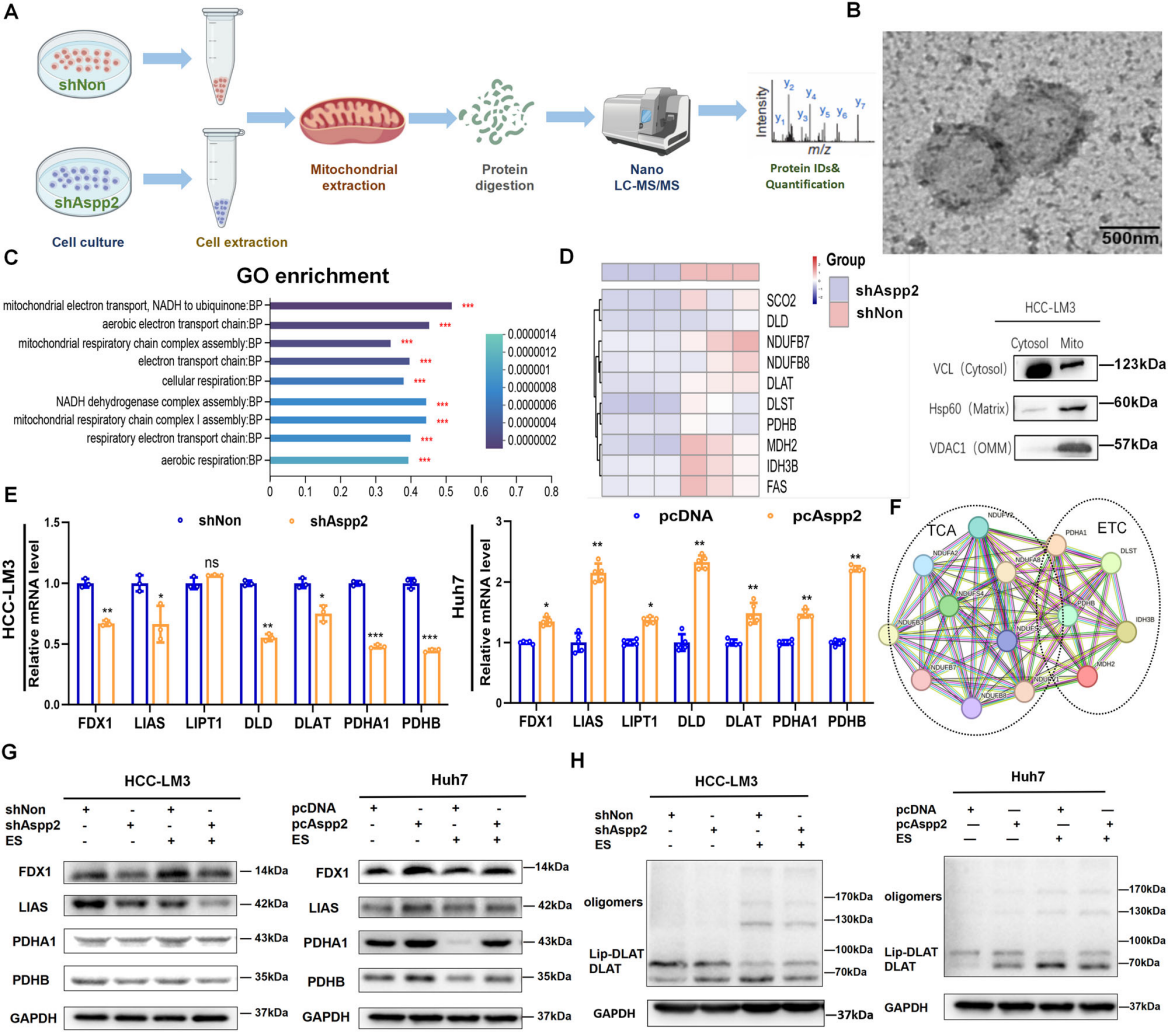
Mitochondrial proteomics revealed the regulatory role of ASPP2 on cuproptosis-related proteins in hepatocellular carcinoma cells. **A** Workflow illustrating mitochondrial extraction and mitochondrial proteomic analysis. **B** TEM images showing the structural integrity of isolated mitochondria with well-defined cristae and double membranes. Western blot analysis confirming mitochondrial enrichment (Hsp60, VDAC1) and VCL. **C** Proteomic profiling of HCC-LM3 cells after ASPP2 knockdown, identifying 679 differentially expressed proteins.GO enrichment analysis highlighting pathways related to the mitochondrial electron transport chain and energy metabolism. **D** Heatmap showing the downregulation of cuproptosis-associated proteins in ASPP2-knockdown HCC-LM3 cells based on proteomic data. **E** qRT-PCR validation of ASPP2-regulated cuproptosis-related genes in HCC-LM3 and Huh7 cells. **F** Protein–protein interaction (PPI) network analysis of ETC and TCA cycle components. **G** Western blot analysis of ASPP2-regulated cuproptosis-related proteins in HCC-LM3 and Huh7 cells. **H** Western blot analysis of lipoylated DLAT (lip-DLAT) in FDX1-overexpressing HCC-LM3 and Huh7 cells treated with ES. Data are expressed as mean ± SD (*n* = 3); *p < 0.05, **p < 0.01, ***p < 0.001.

After isolating mitochondria from HCC-LM3 hepatocellular carcinoma cells we perform mitochondrial proteomes detection (Fig. 3A). The purity of mitochondrial extraction was assessed by immunoblotting and TEM revealed that the mitochondria were round and morphologically intact, with visible double-membrane structures and mitochondrial cristae (Fig. 3B). Cell cytoplasmic proteins were used as controls, and mitochondrial marker enzymes were used to verify the purity of the mitochondrial extract. The results showed that the expression of the mitochondrial reference protein Hsp60 in the mitochondrial protein extract was significantly higher, while the expression of the outer membrane marker enzyme VDAC1 was also notably elevated. The expression of the cytoplasmic protein VCL was significantly lower, confirming that the extracted mitochondria were structurally normal and intact (Fig. 3B).

Next, the mitochondrial proteins were subjected to proteomics analysis to compare protein expression differences after ASPP2 knockdown. A total of 679 differential proteins were identified, with 448 proteins showing decreased expression and 231 proteins showing increased expression after ASPP2 knockdown. Gene Ontology (GO) analysis of genes corresponding to the downregulated proteins revealed that most were associated with the mitochondrial electron transport chain and energy metabolism (***P < 0.0000014) (Fig. 3C and F). Upon further analysis of specific differential genes, several genes involved in the TCA cycle were found, and the expression of multiple genes related to cuproptosis was significantly reduced compared to the control group (*P* < 0.005139) (Fig. 3D). These results suggest that ASPP2 knockdown reduces the expression of cuproptosis-related genes in mitochondria. Cells reliant on mitochondrial respiration are more sensitive to Cuproptosis, implying that ASPP2 may enhance mitochondrial function, thereby increasing the sensitivity of hepatocellular carcinoma cells to Cuproptosis.

FDX1 is an upstream regulator of protein lipidation and encodes a reductase that facilitates the entry of copper ions into cells [30]. This reductase reduces Cu^2+^ to the more toxic Cu+ form, disrupting the cellular copper stability mechanisms and promoting Cuproptosis [31]. LIAS, LIPT1, DLD, and other key genes in the lipidation pathway, including DLAT, PDHA1, and PDHB, can undergo lipidation under the action of FDX1 and other lipidation-related proteins, thereby promoting the occurrence of cuproptosis [32]. Depletion of FDX1 leads to resistance to Cuproptosis in tumor cells. The increased expression of FDX1 and lipidation-related genes suggests a higher probability of cuproptosis in hepatocellular carcinoma cells [33].

In Huh7 hepatocellular carcinoma cells, overexpression of ASPP2 was followed by ES treatment for 72 h. Cells were collected and subjected to real-time quantitative PCR (RT-qPCR) to detect mRNA levels of cuproptosis key enzyme FDX1 and lipidation-related genes. The results showed that, compared to the control group (pcDNA), pcAspp2 overexpression significantly increased the expression of FDX1 and lipidation-related genes (LIAS, LIPT1, DLD, DLAT, PDHA1, PDHB) (*P < 0.05) (Fig. 3E). In HCC-LM3 hepatocellular carcinoma cells, ASPP2 knockdown was performed, followed by ES treatment for 72 h, and cells were collected for RT-qPCR to measure the mRNA levels of the same genes. Compared to the control group (shNon), shAspp2 cells showed a significant reduction in FDX1 and lipidation-related gene expression (*P < 0.05) (Fig. 3E).

In Huh7 hepatocellular carcinoma cells, ASPP2 overexpression followed by ES treatment for 72 h markedly increased the protein levels of FDX1 and lipoylation-related proteins compared with the control group (pcDNA) (Fig. 3G). Conversely, in HCC-LM3 cells, ASPP2 knockdown followed by ES treatment for 72 h significantly reduced the expression of FDX1 and lipoylation-related proteins, including LIAS, PDHA1, and PDHB, relative to the control group (shNon) (Fig. 3G). Notably, ES treatment alone did not cause a significant or consistent change in ASPP2 protein expression in parental Huh7 or HCC-LM3 cells (Fig. S1E). These findings indicate that ASPP2 is not directly induced by ES treatment itself, but rather functions as an upstream determinant that modulates the cellular response to ES-induced cuproptosis-related signaling.

The combined results from RT-qPCR and WB suggest that ASPP2 promotes the expression of cuproptosis key enzymes FDX1, LIAS, PDHA1, and PDHB in hepatocellular carcinoma cells. Protein lipidation is a form of fatty acid modification, where fatty acids are attached to the peptide chain as acyl groups and DBT, GCSH, DLST, and DLAT, which are components of the lipidated TCA cycle enzyme complex, PDHA1 and PDHB [34]. All of these enzymes regulate metabolic complexes that control carbon entry into the TCA cycle, where Cu+ can cause their oligomerization, leading to mitochondrial protein toxicity and triggering cuproptosis [7].

In Huh7 hepatocellular carcinoma cells, overexpression of ASPP2 followed by ES treatment for 72 h resulted in a significant increase in lipidation levels of DLAT, as detected by WB 48 h later. Compared to the control group (pcDNA), the pcAspp2 group showed significant increases in lipidation levels (Fig. 3H right). In HCC-LM3 hepatocellular carcinoma cells, ASPP2 knockdown followed by ES treatment for 72 h resulted in a significant reduction in DLAT lipidation levels 48 hours later, as compared to the control group (shNon) (Fig. 3H left).

### ASPP2 interacts with NR2F2 to regulate FDX1 transcription

Previous studies have shown that genetic ablation of NR2F2 in mouse MA-10 Leydig cells significantly downregulates FDX1 expression, suggesting that NR2F2 functions as a transcriptional activator that directly or indirectly induces FDX1 expression [35]. However, the transcriptional regulatory relationship between NR2F2 and FDX1 remains uncharacterized in human-derived cell lines.

**Fig. 4:**
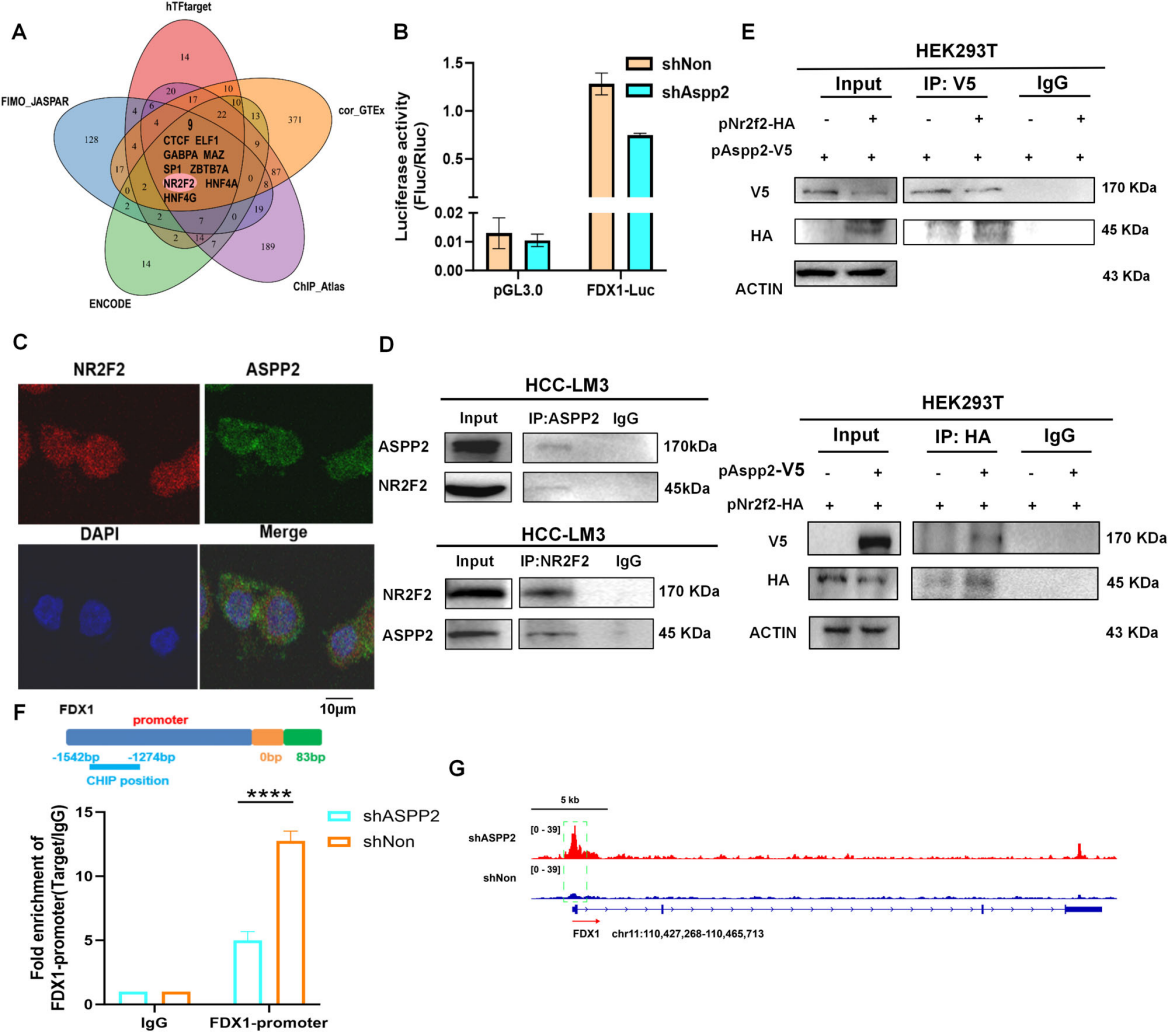
ASPP2 interacts with NR2F2 to regulate FDX1 transcription. **A** Integrated analysis of FITO_JASPAR, ChIP_Atlas, ENCODE, hTFtarget, and cor-GTEx databases predicting potential NR2F2 binding sites within the FDX1 promoter. **B** Dual luciferase reporter assay in HCC-LM3 cells showed that ASPP2 knockdown (LV-shASPP2 vs. LV-shNon) significantly reduced FDX1 promoter activity, confirming ASPP2 as a positive regulator of the FDX1 promoter. **C** Confocal immunofluorescence microscopy displaying nuclear co-localization of ASPP2 and NR2F2 in HCC cells. **D** and **E** Co-IP assays detecting endogenous and exogenous interactions between ASPP2 and NR2F2, and evaluation of NR2F2 transcriptional activity and protein expression upon ASPP2 depletion. **F** Chromatin immunoprecipitation followed by qPCR assessing NR2F2 occupancy at the FDX1 promoter in ASPP2-knockdown and control cells. **G** ATAC-seq analysis showing altered chromatin accessibility at the FDX1 promoter region in ASPP2-silenced (shASPP2) cells compared with shNon cells.

To identify potential transcriptional regulators of FDX1 in HCC, we integrated multiple databases, including JASPAR, ChIPBase, ENCODE, hTFtarget, and cor-GTEx. These analyses predicted that ASPP2 may facilitate the binding of nuclear receptor subfamily 2 group F member 2 (NR2F2) to the promoter region of FDX1 (Fig. 4A).

To functionally validate this regulatory relationship, a dual-luciferase reporter assay was performed in HCC-LM3 cells using an FDX1 promoter reporter construct containing the identified upstream regulatory region. Knockdown of ASPP2 (LV-shASPP2) significantly reduced reporter activity compared with the control group (LV-shNon), indicating that ASPP2 positively regulates FDX1 transcriptional activity (Fig. 4B).

We next examined the spatial relationship between ASPP2 and NR2F2. Confocal immunofluorescence microscopy revealed pronounced nuclear co-localization of endogenous ASPP2 and NR2F2 in HCC cells, supporting a potential functional interaction between these two proteins at the transcriptional level (Fig. 4C). Consistently, co-immunoprecipitation (Co-IP) assays demonstrated both endogenous and exogenous interactions between ASPP2 and NR2F2, confirming that ASPP2 physically associates with NR2F2 (Fig. 4D and E).

Importantly, depletion of ASPP2 did not significantly alter total NR2F2 protein expression; however, it markedly impaired NR2F2 transcriptional activity toward the FDX1 promoter, indicating that ASPP2 functions as a transcriptional co-regulator rather than affecting NR2F2 stability (Figs. 4E and S1F).To determine whether ASPP2 influences NR2F2 binding to the FDX1 promoter, chromatin immunoprecipitation followed by qPCR (ChIP-qPCR) was performed. ASPP2 knockdown significantly reduced NR2F2 occupancy at the FDX1 promoter compared with control cells, demonstrating that ASPP2 is required for efficient NR2F2 recruitment to this region (Fig. 4F). Consistently, ChIP-qPCR analysis presented as percentage of input further showed that ASPP2 depletion markedly decreased enrichment of the −1300 bp distal regulatory region of the FDX1 promoter, confirming ASPP2-dependent binding at this site (Fig. S1G).

Interestingly, ATAC-seq analysis revealed increased chromatin accessibility at the FDX1 promoter in ASPP2-silenced cells relative to shNon controls (Fig. 4G). Although enhanced accessibility is generally permissive for transcription factor binding, the concomitant reduction in NR2F2 occupancy upon ASPP2 loss indicates that ASPP2 is essential for transcription factor recruitment independent of chromatin openness.

Collectively, these results identify ASPP2 as a novel interacting partner and functional co-regulator of NR2F2 that facilitates NR2F2 binding and transcriptional activation of FDX1. The observation that ASPP2 loss increases promoter accessibility while diminishing NR2F2 occupancy highlights a transcription factor-dependent regulatory mechanism rather than a passive chromatin remodeling effect. This ASPP2–NR2F2–FDX1 axis provides mechanistic insight into how ASPP2 promotes FDX1 expression and cuproptosis in HCC.

### ASPP2 promotes cuproptosis through an FDX1-dependent mechanism

**Fig. 5:**
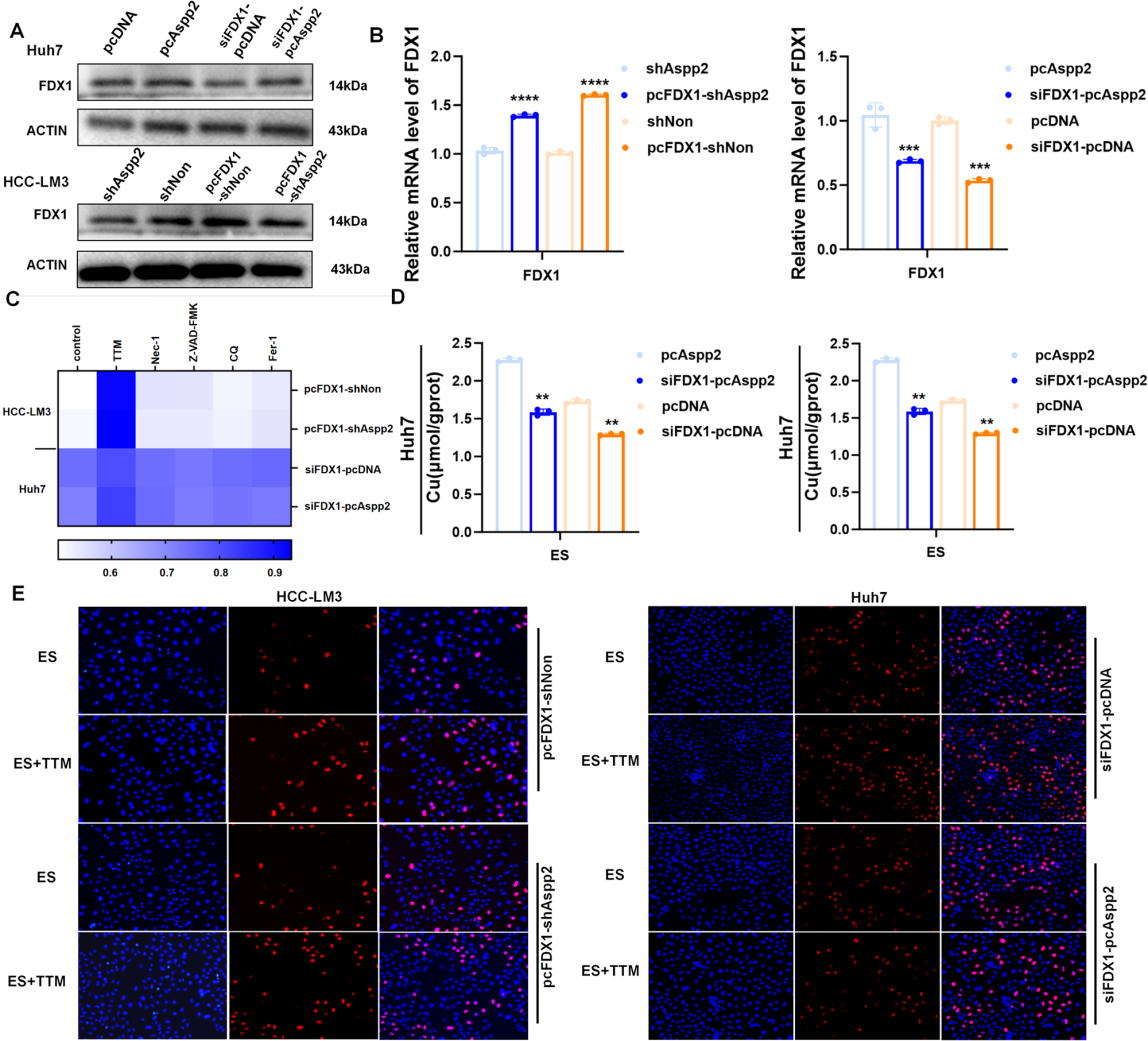
ASPP2 promotes cuproptosis through an FDX1-dependent mechanism. **A** and **B** Overexpression of FDX1 in HCC-LM3 cells with stable ASPP2 knockdown via plasmid transfection and FDX1 silencing in ASPP2-overexpressing Huh7 cells. Transfection efficiency was validated by qRT-PCR and WB. **C** Heatmap analysis of cell death inhibitor screening (Nec-1, CQ, Fer-1, and Z-VAD-FMK) reveals that ES-induced cytotoxicity is mechanistically dependent on FDX1 expression. **D** Quantification of intracellular copper levels in siFDX1-Huh7 and pcFDX1-HCC-LM3 cells after ES treatment. **E** EdU proliferation assay. Scale bar: 100 μm. Data are expressed as mean ± SD (*n* = 3); *p < 0.05, **p < 0.01, ***p < 0.001.

Considering the potential off-target effects of ASPP2 manipulation, we next sought to determine whether the observed phenotypes were mediated through FDX1, a key regulator of protein lipoylation and copper-induced cell death. To this end, we overexpressed FDX1 in HCC-LM3 cells with stable ASPP2 knockdown and silenced FDX1 in Huh7 cells overexpressing ASPP2. The efficiency of FDX1 overexpression and knockdown was confirmed by qPCR and Western blot analyses (Fig. 5A and B).

Cell viability assays using a panel of cell death inhibitors revealed that FDX1 restoration in ASPP2-deficient HCC-LM3 cells markedly reinstated sensitivity to ES-induced cytotoxicity, with ~50% reduction in cell viability compared to controls. Notably, this effect was reversed upon co-treatment with the copper chelator TTM, but not by inhibitors targeting apoptosis, ferroptosis, autophagy, or necroptosis (Fig. 5C). In contrast, silencing FDX1 in ASPP2-overexpressing Huh7 cells abrogated ES-induced growth inhibition, and this insensitivity could not be rescued by TTM, further suggesting a disruption of copper-dependent cytotoxic signaling.

Furthermore, measurements of intracellular copper levels indicated that ASPP2-induced copper accumulation was largely abolished upon FDX1 knockdown, but restored when FDX1 was re-expressed in ASPP2-silenced cells. Collectively, these results demonstrate that ASPP2 facilitates copper-dependent programmed cell death through an FDX1-dependent mechanism (Fig. 5D).

Consistent with these findings, EdU proliferation assays demonstrated that enforced FDX1 expression restored the inhibitory effect of ES in ASPP2-depleted HCC-LM3 cells, while TTM co-treatment reversed this suppression. Conversely, FDX1 knockdown significantly attenuated ES-induced growth inhibition in ASPP2-overexpressing Huh7 cells (Fig. 5E and S1C).

### ASPP2 synergizes with ES–Cu to suppress tumor growth in vivo via cuproptosis

**Fig. 6:**
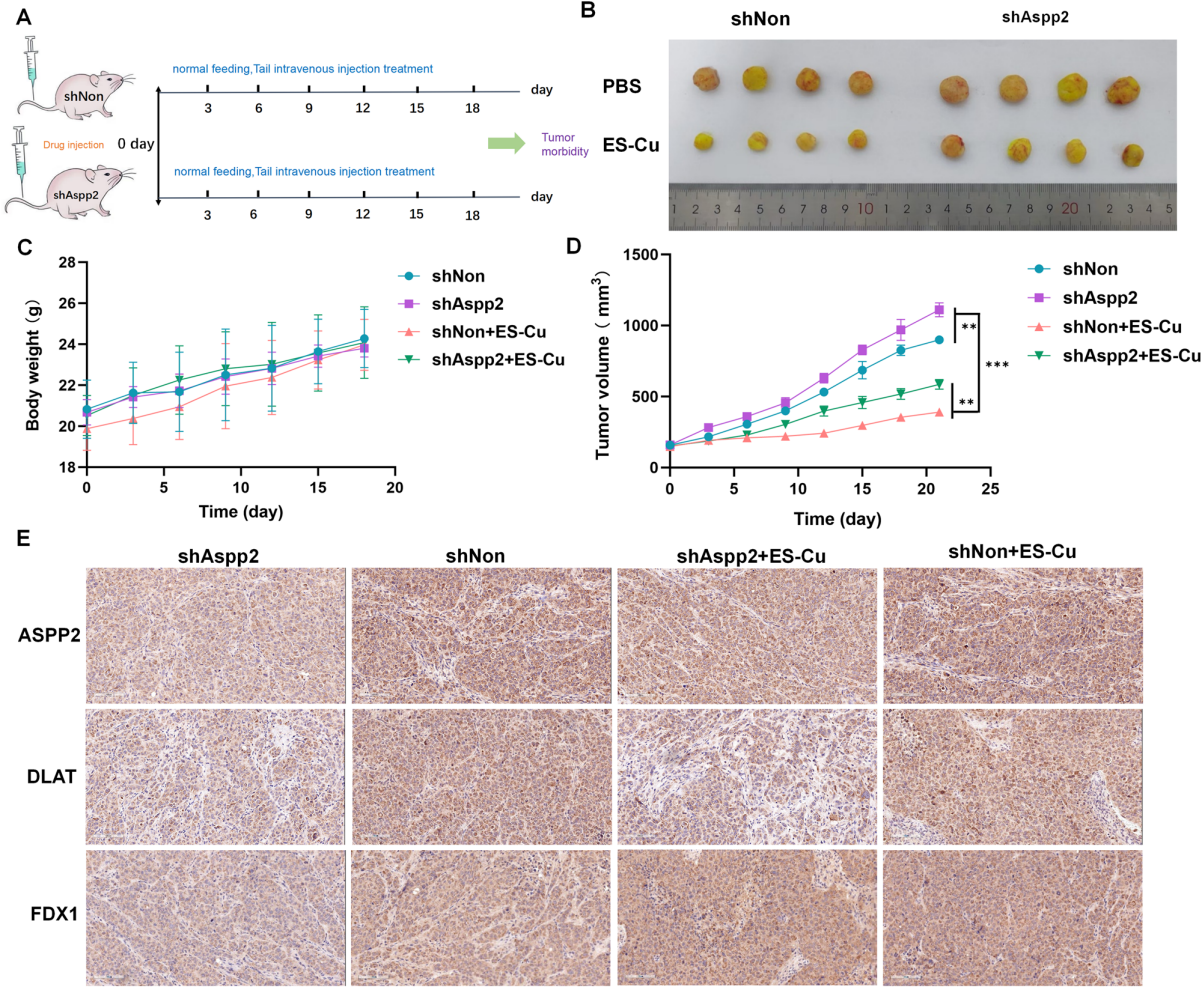
ASPP2 synergizes with ES–Cu to suppress tumor growth in vivo via cuproptosis. **A**–**D** Effects of ES–Cu treatment on tumor growth in xenograft-bearing mice. Mice were administered PBS or ES–Cu intravenously every two days for eight cycles. Tumor volume and body weight were measured during the treatment period and at the endpoint. **P < 0.01, ***P < 0.001 vs. PBS (two-way ANOVA). **E** Representative immunohistochemical staining of ASPP2 and FDX1 in tumor tissues (*n* = 6 per group). Quantitative data are shown as mean ± SD from three independent experiments. Scale bar, 100 μm (magnification, ×400). Statistical significance was assessed using a two-tailed *t*-test.

Due to the existence of cellular copper homeostasis, intracellular copper ions are maintained at limited quantities. ES–Cu can deliver additional exogenous copper to mitochondrial compartments, which has been proven to exhibit promising therapeutic potential in vivo antitumor treatments [36]. We first synthesized ES–Cu by complexing ES with CuCl₂ at a 1:1 ratio. Compared to other copper ionophores, ES–Cu demonstrated superior copper delivery efficiency in vivo. Then we evaluated the antitumor effects of ASPP2 and ES–Cu in a nude mouse model. HCC-LM3 cells expressing either shNon or shAspp2 were subcutaneously injected into the right forelimb of nude mice, with or without ES–Cu treatment. After 21 days of xenograft transplantation, treatment significantly inhibited tumor growth in all groups compared to the control group, with the shNon group showing better therapeutic efficacy than the shAspp2 group (Fig. 6A–D). Furthermore, immunohistochemistry staining revealed that the expression of the cuproptosis gene FDX1 was higher in the shNon group compared to the shAspp2 group (Fig. 6E).

### ASPP2-regulated cuproptosis is associated with tumor progression and provides prognostic value in hepatocellular carcinoma

Emerging evidence has revealed that FDX1 deletion confers resistance to cuproptosis in tumor cells, underscoring its pivotal role in copper-dependent cell death [37]. FDX1 functions as an upstream regulator of protein lipidation, and its activity correlates strongly with components of the lipidation machinery across diverse cell lines [38]. Previous work demonstrated that ASPP2 promotes cuproptosis in HCC by modulating the expression of FDX1 and other lipidation-related genes.

**Fig. 7:**
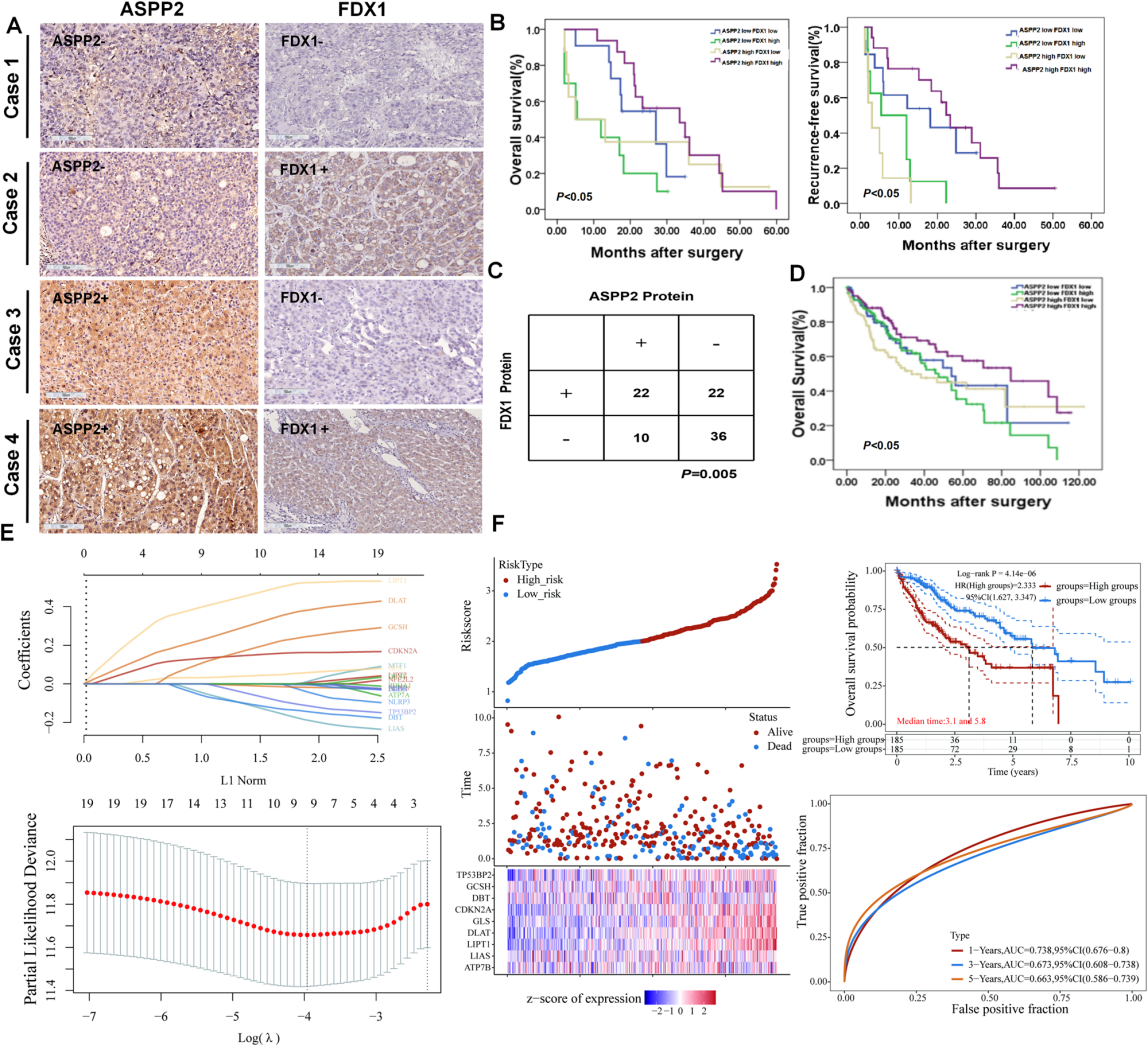
ASPP2-regulated Cuproptosis is associated with tumor progression and provides prognostic value in hepatocellular carcinoma. **A** Immunohistochemical analysis of ASPP2 and FDX1 expression in HCC. **B** and **C** Correlation between ASPP2 and FDX1 protein expression was evaluated by immunohistochemical analysis and adjacent normal tissues and corresponding Kaplan–Meier analysis of OS and RFS. Scale bar: 100 μm. *P* < 0.05 (+: high expression; –: low expression). **D** Kaplan–Meier analysis of OS in TCGA hepatocellular carcinoma patients (*P* < 0.05). **E** Univariate and multivariate Cox regression, combined with Lasso regression, identified cuproptosis key genes associated with prognosis. **F** Time-dependent ROC curves evaluating the predictive performance of the prognostic model for 1-, 3-, and 5-year OS.

To validate these findings in clinical samples, we assessed ASPP2 and FDX1 protein expression in 90 HCC tissues (Fig. 7A). Immunohistochemistry revealed differential expression patterns of ASPP2 and FDX1 between tumor specimens (Fig. 7B; Tables 1–3). The multivariate Cox regression model was globally significant, indicating that the combined variables possess predictive value for patient outcomes. Specifically, low ASPP2 and FDX1 expression was observed in 28.9% of cases, while high expression of both markers occurred in 37.8% (*P* < 0.05). Patients with concurrent high ASPP2 and FDX1 expression exhibited significantly improved overall survival (OS) and recurrence-free survival (RFS) compared with other expression subgroups, suggesting that coactivation of ASPP2 and FDX1 confers a favorable prognosis in HCC (Fig. 7C).

**Table 2:** Univariate analyses of factors associated with recurrence-free survival and overall survival.

	OS		RFS	
Variables	Hazard ratio (95% CI)	*P*	Hazard ratio (95% CI)	*P*
Gender (male vs. female)	0.629(0.236–1.676)	0.354	0.796(0.393–1.611)	0.526
Age, yr (≥50 vs. <50)	0.805(0.384-1.688)	0.566	0.898(0.552–1.460)	0.665
HbsAg (positive vs. negative)	1.219(0.494–3.008)	0.668	1.385(0.656–2.921)	0.393
AFP, ng/mL (>400 vs. ≤400)	2.618(1.233–5.561)	0.012	1.402(0.867–2.268)	0.168
Tumor size, cm (≥5 vs. <5)	1.555(0.732–3.306)	0.251	2.029(1.227–3.355)	0.006
AJCC stage (Ⅲ–Ⅳ vs. Ⅰ–Ⅱ)	2.596(1.104–6.101)	0.029	1.753(1.036–2.965)	0.036
ASPP2 (high vs. low)	0.420(0.192–0.921)	0.03	0.878(0.534–1.442)	0.607
FDX1 (high vs. low)	0.459(0.216–0.974)	0.042	0.940(0.567–1.560)	0.811

**Table 3:** Multivariate analyses of factors associated with overall survival.

	Hazard ratio (95% CI)	*P*
AJCC stage (Ⅲ–Ⅳ vs. Ⅰ–Ⅱ)	2.164(0.853–5.488)	0.104
Tumor size, cm (≥5 vs. <5)	1.591(0.655–3.860)	0.305
ASPP2 (high vs. low)	0.843(0.312–2.279)	0.737
FDX1 (high vs. low)	0.485(0.193–2.618)	0.123

Multivariate analysis, Cox proportional hazards regression model. Variables were adopted for their prognostic significance by univariate analysis and no obvious correlation between them.

In the TCGA cohort (*n* = 470), survival analysis using the Kaplan–Meier method confirmed these associations. RNA-seq values below the median were categorized as low expression, and those above or equal to the median as high. Among four defined subgroups (ASPP2^low^/FDX1^low^, *n* = 69; ASPP2^low^/FDX1^high^, *n* = 124; ASPP2^high^/FDX1^low^, *n* = 105; ASPP2^high^/FDX1^high^, *n* = 105), patients with dual high expression exhibited markedly prolonged OS (*P* < 0.05; Fig. 7D).

A correlation heatmap (Fig. S1D) illustrated distinct expression relationships between ASPP2 and cuproptosis-related genes across three pathological phenotypes. In the “pro-metastatic hepatocyte” group, ASPP2 displayed broad and coherent positive correlations with the core cuproptosis module (FDX1, LIAS, LIPT1/2, DLAT, DLD, PDHA1, PDHB) and copper transport genes (SLC31A1, ATP7A, ATP7B). A similar yet narrower correlation pattern was observed in “pro-tumorigenic hepatocytes,” whereas non-malignant hepatocytes lacked such associations. Control genes (ALB, NLRP3, NFE2L2, GLS, CDKN2A) exhibited no consistent pattern. Although correlation does not establish causality, these results suggest that as HCC progresses, the linkage between ASPP2 and the cuproptosis network strengthens progressively.

Univariate Cox analysis identified several cuproptosis-related genes significantly associated with prognosis (*P* < 0.05). Subsequent multivariate Cox and LASSO regression analyses selected nine key genes—ASPP2, ATP7B, LIAS, LIPT1, DLAT, GLS, CDKN2A, DBT, GCSH, and TP53BP2—to construct a prognostic model (Fig. 7E and F). The final risk score was defined as

Risk score = (−0.0052)×ATP7B + (−0.0808)×LIAS +(0.4324)×LIPT1 + (0.2687)×DLAT + (0.037)×GLS + (0.1412)×CDKN2A + (−0.0858)×DBT + (0.148)×GCSH + (−0.0258)×TP53BP2.

Patients were stratified into high- and low-risk groups according to the median risk score. The high-risk group exhibited significantly higher risk scores and shorter survival durations than the low-risk group, with a hazard ratio (HR) of 2.333 and *P* = 4.14 × 10⁻⁶. ROC analysis showed that the model achieved AUCs of 0.738, 0.673, and 0.663 for predicting 1-, 3-, and 5-year OS, respectively, indicating moderate predictive accuracy.

To assess external validity, the same risk score formula was applied to the independent ICGC cohort (Fig. S1H). Risk distribution and survival status plots demonstrated that mortality increased with higher risk scores, paralleling the TCGA results. Kaplan–Meier analysis revealed that patients in the high-risk group had significantly worse survival (log-rank *P* = 0.000266; HR = 3.614; 95% CI: 1.812–7.211), with a median OS of 3.5 years. Heatmap visualization confirmed that DLAT and CDKN2A were upregulated in high-risk cases, consistent with their positive regression coefficients. The time-dependent ROC curves yielded AUCs of 0.661 (95% CI: 0.513–0.809), 0.785 (95% CI: 0.673–0.843), and 0.325 (95% CI: 0.160–0.489) for 1-, 3-, and 5-year OS, respectively, indicating robust short- and intermediate-term predictive power. Collectively, these findings demonstrate that ASPP2-regulated cuproptosis is intricately linked to HCC progression and that the ASPP2–cuproptosis gene signature provides a stable and clinically meaningful prognostic tool validated across independent cohorts.

## Discussion and conclusion

Our study demonstrates that ASPP2 plays a pivotal role in regulating mitochondrial function and enhancing the susceptibility of HCC cells to cuproptosis. Through an integrative approach combining proteomic, functional, and bioinformatic analyses, we reveal that ASPP2 promotes mitochondrial OXPHOS while suppressing aerobic glycolysis, thereby orchestrating a metabolic shift that increases cellular dependence on mitochondrial respiration [39]. Given the established link between mitochondrial respiration and cuproptosis sensitivity, our findings identify ASPP2 as a key regulator of this novel cell death pathway in HCC [40].

**Fig. 8:**
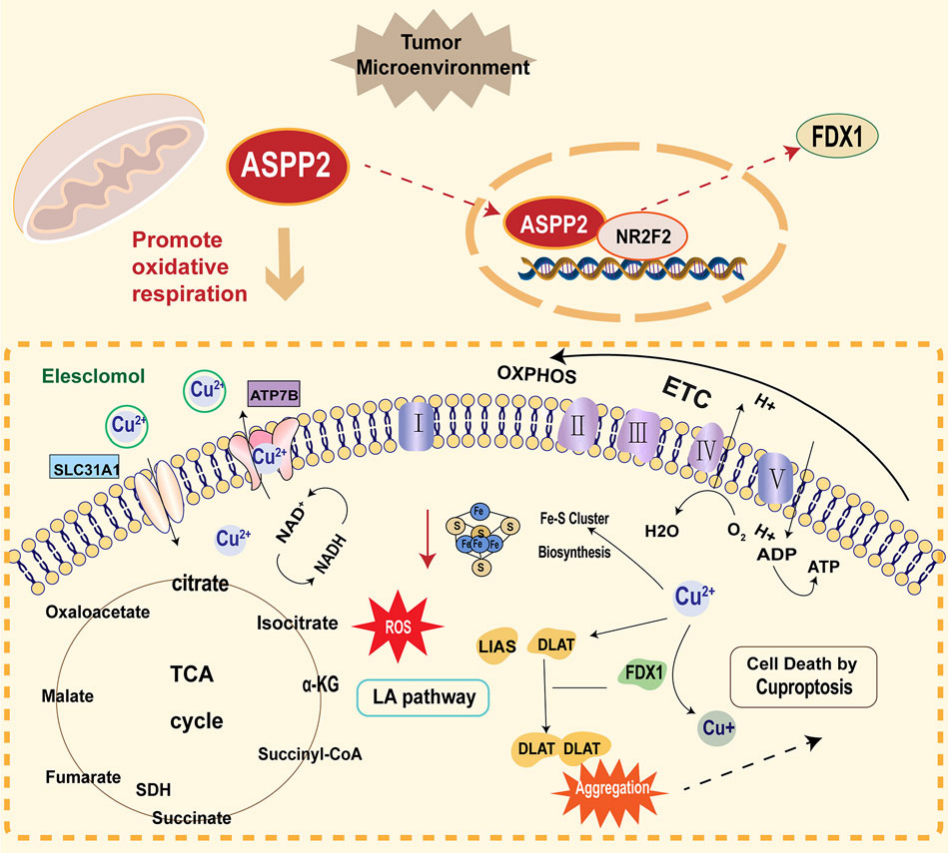
Schematic representation of the mechanism of ASPP2-regulated mitochondrial function in promoting cuproptosis in hepatocellular carcinoma cells. ASPP2 plays a crucial role in regulating mitochondrial function to facilitate cuproptosis in HCC cells. It inhibits aerobic glycolysis while enhancing mitochondrial respiration and the expression of key enzymes in the TCA cycle. Moreover, ASPP2 upregulates the expression of FDX1, a pivotal regulator of cuproptosis, thereby promoting downstream lipoylation processes and increasing the lipoylation levels of mitochondrial proteins. The FDX1 accelerates the reduction of Cu²⁺, delivered by elesclomol, to Cu⁺. The accumulation of Cu⁺ leads to its binding with lipoylated proteins, inducing their oligomerization. Concurrently, Cu⁺ disrupts iron-sulfur cluster proteins, leading to proteotoxic stress and mitochondrial dysfunction. This cascade ultimately triggers cuproptosis, highlighting ASPP2 as a critical regulator of mitochondrial metabolism and copper-dependent cell death in HCC.

Mechanistically, ASPP2 enhances the expression and activity of key TCA cycle enzymes, including PDH, CS, and α-KGDH, thereby bolstering mitochondrial respiration. Proteomic profiling of ASPP2-depleted HCC cells reveals a significant downregulation of mitochondrial proteins implicated in copper metabolism and cuproptosis. FDX1 is the upstream regulator of protein lipoylation. FDX1 catalyzes the reduction of Cu²⁺ to Cu⁺, a critical step in driving lipoylation-dependent protein aggregation and subsequent proteotoxic stress. Our data suggest that ASPP2 regulates FDX1 expression to facilitate cuproptosis, a notion further supported by the concomitant reduction in lipoylation-related proteins (LIAS, LIPT1, PDHA1, and PDHB) upon ASPP2 knockdown (Fig. 8).

Expanding on these findings, we employed multiple databases (JASPAR, CHIPbase, ENCODE, hTFtarget, and cor-GTEX) to predict potential transcription factor binding sites, revealing that ASPP2 may promote the binding of NR2F2 to the promoter region of FDX1 in HCC cells. Subsequent ChIP-qPCR experiments confirmed that ASPP2 enhances the recruitment of NR2F2 to the FDX1 promoter, while Co-IP and IF assays validated their physical interaction. This transcriptional regulation underscores a novel ASPP2–FDX1–NR2F2 axis, which transcriptionally activates FDX1 and promotes mitochondrial lipoylation and cuproptosis.

Functionally, pharmacological studies reveal that ASPP2-overexpressing HCC cells are significantly more sensitive to ES, a copper ionophore known to trigger cuproptosis. This sensitization can be rescued by TTM, a copper chelator, confirming the dependence on intracellular copper. Notably, inhibitors of Nec-1, Fer-1, Z-VAD-FMK, and CQ fail to prevent ES-induced cytotoxicity, indicating that ASPP2-mediated cell death is specific to the cuproptosis pathway.

Clinically, analysis of TCGA data and 90 hepatocellular carcinoma tissue samples reveals a strong positive correlation between ASPP2 and FDX1 expression in HCC tissues. Patients with high ASPP2/FDX1 expression exhibit lower tumor stages, improved OS, and prolonged RFS, suggesting that the ASPP2–FDX1 axis may serve as a robust prognostic biomarker. Moreover, a prognostic model integrating ASPP2 with cuproptosis-related genes outperforms models based solely on cuproptosis genes, as evidenced by higher HR and AUC values. Kaplan–Meier analysis further corroborates that dual high expression of ASPP2 and FDX1 predicts the most favorable clinical outcomes, highlighting the translational potential of this pathway.

In conclusion, our study identifies ASPP2 as a master regulator of mitochondrial metabolism and cuproptosis in HCC. By promoting NR2F2-mediated transcriptional activation of FDX1 and enhancing mitochondrial OXPHOS, ASPP2 establishes a metabolic context that heightens vulnerability to copper-induced cell death (Fig. 8). These findings not only elucidate a novel mechanism linking tumor suppressor function to metabolic stress responses but also provide a compelling rationale for the development of ASPP2-targeted therapies in combination with copper ionophores for the treatment of HCC.

## Supplementary information

Supplementary Material

Supplementary Material-W B

## Data Availability

All remaining data are available within the article and supplementary files, or available from the authors upon request.

## References

[1] Llovet JM, Kelley RK, Villanueva A, Singal AG, Pikarsky E, Roayaie S, et al. Hepatocellular carcinoma[J]. Nat Rev Dis Primers. 2021;7:6. 33479224 10.1038/s41572-020-00240-3 PMC13537433

[2] Yang C, Zhang H, Zhang L, Zhu AX, Bernards R, Qin W, et al. Evolving therapeutic landscape of advanced hepatocellular carcinoma[J]. Nat Rev Gastroenterol Hepatol. 2023;20:203–222. 36369487 10.1038/s41575-022-00704-9

[3] Singal AG, Kanwal F, Llovet JM. Global trends in hepatocellular carcinoma epidemiology: implications for screening, prevention and therapy[J]. Nat Rev Clin Oncol. 2023;20:864–884. 37884736 10.1038/s41571-023-00825-3

[4] Ladd AD, Duarte S, Sahin I, Zarrinpar A. Mechanisms of drug resistance in HCC[J]. Hepatology. 2024;79:926–940. 36680397 10.1097/HEP.0000000000000237

[5] Llovet JM, Castet F, Heikenwalder M, Maini MK, Mazzaferro V, Pinato DJ, et al. Immunotherapies for hepatocellular carcinoma[J]. Nat Rev Clin Oncol. 2022;19:151–172. 34764464 10.1038/s41571-021-00573-2

[6] Wang Y, Patti GJ. The Warburg effect: a signature of mitochondrial overload[J]. Trends Cell Biol. 2023;33:1014–1020. 37117116 10.1016/j.tcb.2023.03.013 PMC10600323

[7] Tsvetkov P, Coy S, Petrova B, Dreishpoon M, Verma A, Abdusamad M, et al. Copper induces cell death by targeting lipoylated TCA cycle proteins[J]. Science. 2022;375:1254–1261. 35298263 10.1126/science.abf0529 PMC9273333

[8] Tsvetkov P, Detappe A, Cai K, Keys HR, Brune Z, Ying W, et al. Mitochondrial metabolism promotes adaptation to proteotoxic stress[J]. Nat Chem Biol. 2019;15:681–689. 31133756 10.1038/s41589-019-0291-9 PMC8183600

[9] Chen L, Min J, Wang F. Copper homeostasis and cuproptosis in health and disease[J]. Signal Transduct Target Ther. 2022;7:378. 36414625 10.1038/s41392-022-01229-y PMC9681860

[10] Cobine PA, Brady DC. Cuproptosis: cellular and molecular mechanisms underlying copper-induced cell death[J]. Mol Cell. 2022;82:1786–1787. 35594843 10.1016/j.molcel.2022.05.001

[11] Tang D, Kroemer G, Kang R. Targeting cuproplasia and cuproptosis in cancer[J]. Nat Rev Clin Oncol. 2024;21:370–388. 38486054 10.1038/s41571-024-00876-0

[12] Lin CH, Chin Y, Zhou M, Sobol RW, Hung MC, Tan M. Protein lipoylation: mitochondria, cuproptosis, and beyond[J]. Trends Biochem Sci. 2024;49:729–744. 38714376 10.1016/j.tibs.2024.04.002

[13] Liao Q, Deng J, Tong J, Gan Y, Hong W, Dong H, et al. p53 induces circFRMD4A to suppress cancer development through glycolytic reprogramming and cuproptosis[J]. Mol Cell. 2025;85:132–149.e7. 39637854 10.1016/j.molcel.2024.11.013

[14] Xie J, Yang Y, Gao Y, He J. Cuproptosis: mechanisms and links with cancers[J]. Mol Cancer. 2023;22:46. 36882769 10.1186/s12943-023-01732-y PMC9990368

[15] Xue Q, Kang R, Klionsky DJ, Tang D, Liu J, Chen X. Copper metabolism in cell death and autophagy[J]. Autophagy. 2023;19:2175–2195. 37055935 10.1080/15548627.2023.2200554 PMC10351475

[16] Wang Y, Chen Y, Zhang J, Yang Y, Fleishman JS, Wang Y, et al. Cuproptosis: a novel therapeutic target for overcoming cancer drug resistance[J]. Drug Resist Updat. 2024;72:101018. 37979442 10.1016/j.drup.2023.101018

[17] Vives V, Su J, Zhong S, Ratnayaka I, Slee E, Goldin R, et al. ASPP2 is a haploinsufficient tumor suppressor that cooperates with p53 to suppress tumor growth[J]. Genes Dev. 2006;20:1262–7. 16702401 10.1101/gad.374006 PMC1472901

[18] Zak J, Vives V, Szumska D, Vernet A, Schneider JE, Miller P, et al. ASPP2 deficiency causes features of 1q41q42 microdeletion syndrome[J]. Cell Death Differ. 2016;23:1973–1984. 27447114 10.1038/cdd.2016.76 PMC5136487

[19] Liang B, Jiang Y, Song S, Jing W, Yang H, Zhao L, et al. ASPP2 suppresses tumour growth and stemness characteristics in HCC by inhibiting Warburg effect via WNT/β-catenin/HK2 axis[J]. J Cell Mol Med. 2023;27:659–671. 36752127 10.1111/jcmm.17687 PMC9983321

[20] Chen R, Wang H, Liang B, Liu G, Tang M, Jia R, et al. Downregulation of ASPP2 improves hepatocellular carcinoma cells survival via promoting BECN1-dependent autophagy initiation[J]. Cell Death Dis. 2016;7:e2512. 27929538 10.1038/cddis.2016.407 PMC5260975

[21] Liang B, Chen R, Song S, Wang H, Sun G, Yang H, et al. ASPP2 inhibits tumor growth by repressing the mevalonate pathway in hepatocellular carcinoma[J]. Cell Death Dis. 2019;10:830. 31685796 10.1038/s41419-019-2054-7 PMC6828733

[22] Lu Y, Yang A, Quan C, Pan Y, Zhang H, Li Y, et al. A single-cell atlas of the multicellular ecosystem of primary and metastatic hepatocellular carcinoma. Nat Commun. 2022;13:4594. 35933472 10.1038/s41467-022-32283-3 PMC9357016

[23] Zhao J, Wu G, Bu F, Lu B, Liang A, Cao L, et al. Epigenetic silence of ankyrin-repeat-containing, SH3-domain-containing, and proline-rich-region-containing protein 1 (ASPP1) and ASPP2 genes promotes tumor growth in hepatitis B virus-positive hepatocellular carcinoma[J]. Hepatology. 2010;51:142–53. 20034025 10.1002/hep.23247

[24] Sainero-Alcolado L, Liaño-Pons J, Ruiz-Pérez MV, Arsenian-Henriksson M. Targeting mitochondrial metabolism for precision medicine in cancer[J]. Cell Death Differ. 2022;29:1304–1317. 35831624 10.1038/s41418-022-01022-y PMC9287557

[25] Lee KM, Giltnane JM, Balko JM, Schwarz LJ, Guerrero-Zotano AL, Hutchinson KE, et al. MYC and MCL1 cooperatively promote chemotherapy-resistant breast cancer stem cells via regulation of mitochondrial oxidative phosphorylation[J]. Cell Metab. 2017;26:633–647.e7. 28978427 10.1016/j.cmet.2017.09.009 PMC5650077

[26] Yuan W, Lu G, Zhao Y, He X, Liao S, Wang Z, et al. Intranuclear TCA and mitochondrial overload: The nascent sprout of tumors metabolism[J]. Cancer Lett. 2025;613:217527. 39909232 10.1016/j.canlet.2025.217527

[27] Cui L, Gouw AM, LaGory EL, Guo S, Attarwala N, Tang Y, et al. Mitochondrial copper depletion suppresses triple-negative breast cancer in mice[J]. Nat Biotechnol. 2021;39:357–367. 33077961 10.1038/s41587-020-0707-9 PMC7956242

[28] Zhang M, Qiu H, Mao L, Wang B, Li N, Fan Y, et al. Ammonium tetrathiomolybdate triggers autophagy-dependent NRF2 activation in vascular endothelial cells[J]. Cell Death Dis. 2022;13:733. 36008391 10.1038/s41419-022-05183-z PMC9411162

[29] Kim YJ, Tsang T, Anderson GR, Posimo JM, Brady DC. Inhibition of BCL2 family members increases the efficacy of copper chelation in BRAF(V600E)-driven melanoma[J]. Cancer Res. 2020;80:1387–1400. 32005716 10.1158/0008-5472.CAN-19-1784 PMC7127963

[30] Feng S, Zhang Y, Zhu H, Jian Z, Zeng Z, Ye Y, et al. Cuproptosis facilitates immune activation but promotes immune escape, and a machine learning-based cuproptosis-related signature is identified for predicting prognosis and immunotherapy response of gliomas[J]. CNS Neurosci Ther. 2024;30:e14380. 37515314 10.1111/cns.14380 PMC10848101

[31] Sun Z, Xu H, Lu G, Yang C, Gao X, Zhang J, et al. AKT1 Phosphorylates FDX1 to Promote Cuproptosis Resistance in Triple-Negative Breast Cancer. Adv Sci (Weinh). 2025;12:e2408106. 39976173 10.1002/advs.202408106 PMC12061301

[32] Schulz V, Basu S, Freibert SA, Webert H, Boss L, Mühlenhoff U, et al. Functional spectrum and specificity of mitochondrial ferredoxins FDX1 and FDX2[J]. Nat Chem Biol. 2023;19:206–217. 36280795 10.1038/s41589-022-01159-4 PMC10873809

[33] Zulkifli M, Spelbring AN, Zhang Y, Soma S, Chen S, Li L, et al. FDX1-dependent and independent mechanisms of elesclomol-mediated intracellular copper delivery[J]. Proc Natl Acad Sci USA. 2023;120:e2216722120. 36848556 10.1073/pnas.2216722120 PMC10013847

[34] Zhu S, Niu Y, Zhou W, Liu Y, Liu J, Liu X, et al. Mitochondrial copper overload promotes renal fibrosis via inhibiting pyruvate dehydrogenase activity[J]. Cell Mol Life Sci. 2024;81:340. 39120696 10.1007/s00018-024-05358-1 PMC11335263

[35] Mehanovic S, Mendoza-Villarroel RE, de Mattos K, Talbot P, Viger RS, Tremblay JJ. Identification of novel genes and pathways regulated by the orphan nuclear receptor COUP-TFII in mouse MA-10 Leydig cells†[J]. Biol Reprod. 2021;105:1283–1306. 34225363 10.1093/biolre/ioab131 PMC8599028

[36] Guo B, Yang F, Zhang L, Zhao Q, Wang W, Yin L, et al. Cuproptosis induced by ROS responsive nanoparticles with elesclomol and copper combined with αPD-L1 for enhanced cancer immunotherapy[J]. Adv Mater. 2023;35:e2212267. 36916030 10.1002/adma.202212267

[37] Wang W, Lu K, Jiang X, Wei Q, Zhu L, Wang X, et al. Ferroptosis inducers enhanced cuproptosis induced by copper ionophores in primary hepatocellular carcinoma [J]. J Exp Clin Cancer Res. 2023;42:142. 37277863 10.1186/s13046-023-02720-2 PMC10242978

[38] Sun L, Zhang Y, Yang B, Sun S, Zhang P, Luo Z, et al. Lactylation of METTL16 promotes cuproptosis via m(6)A-modification on FDX1 mRNA in gastric cancer[J]. Nat Commun. 2023;14:6523. 37863889 10.1038/s41467-023-42025-8 PMC10589265

[39] Xiong C, Ling H, Hao Q, Zhou X. Cuproptosis: p53-regulated metabolic cell death?[J]. Cell Death Differ. 2023;30:876–884. 36755067 10.1038/s41418-023-01125-0 PMC10070433

[40] Shao N, Yang Y, Hu G, Luo Q, Cheng N, Chen J, et al. Synergistic enhancement of low-dose radiation therapy via cuproptosis and metabolic reprogramming for radiosensitization in in situ hepatocellular carcinoma[J]. J Nanobiotechnol. 2024;22:772. 10.1186/s12951-024-03011-4 PMC11657429 39696547

