# Supplementary material for: ASPP2 inhibits hepatocellular carcinoma growth by regulating cuproptosis via mitochondrial protein lipoylation
Source: Cell Death Dis. 2026 Apr 24;17(1):685. doi: 10.1038/s41419-026-08704-2 (PMC13443132; doi:10.1038/s41419-026-08704-2)
Supplement: Supplementary file 1 [file 41419_2026_8704_MOESM1_ESM.docx]

# **Supplementary Material**

## Supplementary Figures

**Figure S1**

(A-B) qPCR and western blot analyses verifying ASPP2 overexpression efficiency in Huh7 and HCC-LM3 cells transfected with pcASPP2 or pcDNA. Relative mRNA and protein levels are normalized to β-actin. Data are presented as mean ± SEM from three independent experiments (P < 0.01, P < 0.001).

(C)Quantification of EdU incorporation assays assessing proliferative activity in Huh7 and HCC-LM3 cells after FDX1 overexpression or knockdown. Data are shown as mean ± SEM from three independent experiments; significance was determined by Student’s t-test (P < 0.001).

(D)Correlation heatmap showing the relationships between ASPP2 and cuproptosis-related or control genes at the single-cell level across three hepatocyte pathological phenotypes.

(E) Western blot analysis of ASPP2 protein levels in parental Huh7 and HCC-LM3 cells treated with ES (+) or vehicle control (-), with ACTIN as a loading control.

(F)Western blot analysis of NR2F2 protein expression in HCC-LM3 cells with ASPP2 knockdown (shAspp2) and control (shNon) groups, using ACTIN as a loading control.

(G)ChIP-qPCR results presented as % of input, comparing enrichment at the FDX1 promoter -1300 bp region in HCC-LM3 cells with ASPP2 knockdown (shASPP2) and control (shNon) groups.

(H)External validation of the ASPP2-cuproptosis prognostic signature in the ICGC cohort. Univariate and multivariate Cox regression combined with LASSO analysis were performed to identify prognostic genes. Risk groups were defined according to the median risk score. Time-dependent ROC curves were generated to evaluate 1-, 3-, and 5-year overall-survival predictive performance.

**Table S1. Primer sequences are listed below**

| Primer |  | sequences 5’-3’ |
| --- | --- | --- |
| β-actin | F | TGTTACCAACTGGGACGACA |
|  | R | CTGGGTCATCTTTTCACGGT |
| ASPP2 | F | GAAGACTCGGTGAGCATGCG |
|  | R | GCGATACGCTCTGAGCCAGT |
| FDX1 | F | CACCGGTCCACTTTATAAACCGTGA |
|  | R | AAACTCACGGTTTATAAAGTGGACC |
| LIAS | F | CACCGCAGGAGTAAGACACTCCACA |
|  | R | AAACTGTGGAGTGTCTTACTCCTGC |
| DLAT | F | GCGACGGGCTCAGAATGTAG |
|  | R | CGCCATACCCTGTAGTCACG |
| PDHA1 | F | TGGTAGCATCCCGTAATTTTGC |
|  | R | ATTCGGCGTACAGTCTGCATC |
| PDHB | F | GTTGCGGCCCATTTGTGAAT |
|  | R | ATACCAGGCAGCAAAGCACT |
| LIPT1 | F | CACCGATGCCTACCAATTACAACAG |
|  | R | AAACCTGTTGTAATTGGTAGGCATC |

**Table S2. Chip-qPCR primer sequence**

| **primer** | | **sequence（5'-3'）** | | |
| --- | --- | --- | --- | --- |
|  | HsproFDX1<-NR2F2-chip-S | | CTGGAGGGTTGGTAAAGGCC |  |
|  | HsproFDX1<-NR2F2-chip-A | | TTGGAGTCTCTCGCGGCC |  |
|  | HsproFDX1<-NR2F2-chip-F | | GAAACTCTGATCCTCCATGAGAA |  |
|  | HsproFDX1<-NR2F2-chip-R | | CATCCTAGACCTTGACTGGCA |  |
|  | Hs-Gapdh-F | | GGAGCGAGATCCCTCCAAAAT |  |
|  | Hs-Gapdh-R | | GGCTGTTGTCATACTTCTCATGG |  |
