# Supplementary material for: ASPP2 inhibits hepatocellular carcinoma growth by regulating cuproptosis via mitochondrial protein lipoylation
Source: Cell Death Dis. 2026 Apr 24;17(1):685. doi: 10.1038/s41419-026-08704-2 (PMC13443132; doi:10.1038/s41419-026-08704-2)
Supplement: Supplementary file 2 [file 41419_2026_8704_MOESM2_ESM.docx]

**Figure 3G**

**HCC-LM3:**

**Huh7:**

**Figure 3D**

LM3-HCC

LIAS

**Figure 4E**

**HEK293T**

**ASPP2-NR2F2**

HEK293T

IP: V5

V5

HEK293T

IP: V5

HA

HEK293T

IP: V5

actin

**NR2F2-ASPP2**

HEK293T

IP: HA

V5

HEK293T

IP: HA

V5

HEK293T

IP: HA

HA

HEK293T

IP: HA

HA

HEK293T

IP: HA

actin

**Figure 4F**

**ASPP2**

**Input**

**IP+IgG**

**ASPP2**

**Figure 5A**

**Figure S1A**

100 KDa

**Figure S1B**

**Figure S1C**

**Figure S1**
